# A case study of a collision tumor composed of cancers of the bile duct and pancreas

**DOI:** 10.1186/s40792-015-0041-5

**Published:** 2015-05-09

**Authors:** Hideki Izumi, Daisuke Furukawa, Naoki Yazawa, Yoshihito Masuoka, Misuzu Yamada, Kosuke Tobita, Yohei Kawashima, Masami Ogawa, Yoshiaki Kawaguchi, Kenichi Hirabayashi, Toshio Nakagohri

**Affiliations:** Department of Gastrointestinal Surgery, Tokai University School of Medicine, 143 Shimokasuya, Isehara, Kanagawa 259-1193 Japan; Department of Internal Medicine, Tokai University School of Medicine, 143 Shimokasuya, Isehara, Kanagawa 259-1193 Japan; Department of Pathology, Tokai University School of Medicine, 143 Shimokasuya, Isehara, Kanagawa 259-1193 Japan

**Keywords:** Collision tumor, Bile duct cancer, Pancreatic cancer

## Abstract

In this case report, we describe the extremely rare case of a collision tumor comprising cancers of the bile duct and the pancreas. A 70-year-old man was referred to our hospital with a diagnosis of obstructive jaundice. He was diagnosed with pancreatic head cancer, and we performed a pancreaticoduodenectomy with lymph node dissection. At laparotomy, there were two palpable masses in the vicinity of the confluence of the cystic duct and the head of the pancreas. The resected specimen demonstrated tumors at the confluence of the cystic duct and in the pancreatic head. Histopathological examination demonstrated a moderately differentiated tubular adenocarcinoma in the pancreatic head and a well-differentiated tubular adenocarcinoma at the confluence of the cystic duct. Immunostaining was negative for p53 and MUC6 in the pancreatic head tumor; however, immunostaining was positive for both in the tumor located at the confluence of the cystic duct. The two tumors were histologically different and were diagnosed as collision cancer caused by the collision of the bile duct and pancreatic cancers.

## Background

Collision cancers are malignancies in the same organ or anatomical site that comprises at least two different tumor components, with no mixed or transitional area between two components. Dual cancer involving a combination of pancreatic and bile duct cancers is rare, and to our knowledge, few cases of a collision cancer composed of these two specific cancers have been previously reported [1]. We encountered a patient with a very rare collision cancer in which the pancreatic and bile duct cancers had collided.

## Case presentation

### Patient

Patient was a 70-year-old male.

### Chief complaint

Chief complaint was right upper abdominal pain.

### Past medical history

Past medical history was unremarkable.

### Family medical history

Family medical history was unremarkable.

### History of present illness

The patient presented to his physician with a chief complaint of right upper abdominal pain in November 2009. Blood testing demonstrated obstructive jaundice, and the patient was admitted to our hospital for further evaluation.

### Relevant physical exam

The palpebral conjunctivae were mildly yellow, but no pallor was noted. The abdomen was flat and soft without tenderness, and no mass was palpated.

Blood test findings on admission were as follows (Table 1): aspartate aminotransferase (AST), 179 IU/L; alanine transaminase (ALT), 244 IU/L; alkaline phosphatase (ALP), 2,097 IU/L; and γ-glutamyltransferase (γ-GTP), 1,246 IU/L. Thus, serum levels of hepatobiliary enzymes were elevated. Serum level of T-Bil was 2.7 mg/dL and that of D-Bil was 1.5 mg/dL, demonstrating mild jaundice. Moreover, tumor marker elevation was observed: serum level of CEA was 13.0 ng/mL and that of CA19-9 was 515.3 U/mL.

**Table 1:** **Laboratory data on admission**

	**Value**
WBC	8.4 × 10^3^/μL
RBC	4.56 × 10^6^/μL
Hb	14.0 g/dL
Ht	41.5%
PLT	18.9 × 10^4^/μL
BUN	13 mg/dL
Cr	0.7 mg/dL
Na	139 mEq/L
K	4.5 mEq/L
Cl	101 mEq/L
Ca	9.7 mg/dL
CRP	2.22 mg/dL
ALB	3.7 g/dL
CK	40 IU/L
AST	179 IU/L
ALT	244 IU/L
ALP	2,097 IU/L
γ-GTP	1,246 IU/L
T-Bil	2.5 mg/dL
D-Bil	1.5 mg/dL
AMY	29 IU/L
CEA	13.0 ng/mL
CA19-9	515.3 U/mL

### Abdominal ultrasonography

An approximately 3-cm, low-echoic mass with unclear boundaries and heterogeneous content was detected in the pancreatic head. There was a 17.8-mm dilation of the common bile duct and a 2.9-mm dilation of the caudal pancreatic duct due to the mass.

### Abdominal computed tomography

**Figure 1:**
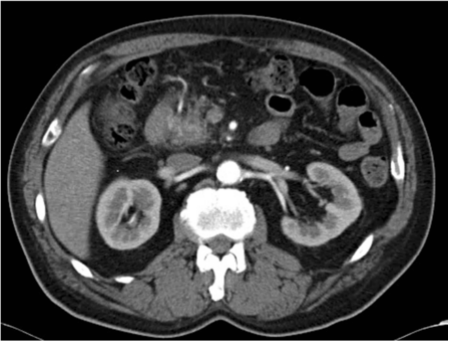
The tumor boundary within the pancreatic head is unclear in the non-uniform contrast equilibrium phase-arterial phase.

There was no clearly visible mass. A mild enhancement of the intrapancreatic common bile duct wall in both the arterial and the equilibrium phases was detected (Figure 1). The intrahepatic bile ducts were dilated bilaterally, and there was mild dilation of the pancreatic duct.

### Magnetic resonance cholangiopancreatography

The intrapancreatic bile duct tapered off sharply, and there was marked dilation of the duct proximal to this obstruction.

### Endoscopic retrograde cholangiopancreatography

**Figure 2:**
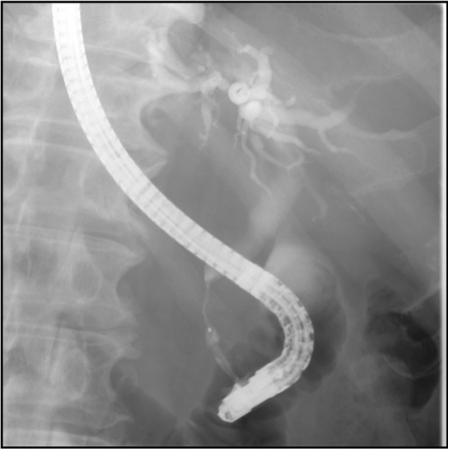
Cholangiographic examination shows severe stenosis of the lower bile duct.

Stenosis of the lower bile duct extending over approximately 2 cm was noted (Figure 2). An endoscopic retrograde biliary drainage tube was inserted. The pancreatic duct could not be cannulated. Bile cytology was class V. Because of these findings, the patient was preoperatively diagnosed with pancreatic head cancer accompanied by bile duct infiltration, and pylorus-preserving pancreaticoduodenectomy was performed.

### Surgical findings

At laparotomy, the abdomen was opened using an upper abdominal midline incision. No ascites or peritoneal seeding was noted. There were two palpable masses in the vicinity of the confluence of the cystic duct and the head of the pancreas. The resected margins of the pancreatic and biliary ducts were subjected to rapid intraoperative pathological examination, and the bile duct remnant was found to be positive for residual tumor cells. After additional resection, rapid intraoperative pathological examination confirmed clear margins. Pylorus-preserving pancreaticoduodenectomy and lymph node dissection were performed.

### Pathological findings

**Figure 3:**
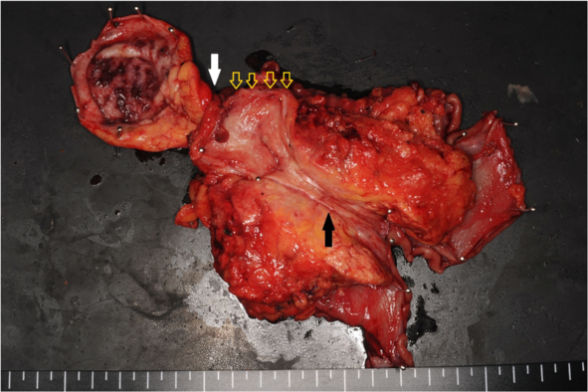
The resected specimen demonstrated tumors in both the bile duct and the pancreatic head. Stenosis due to bile duct invasion by pancreatic head cancer was noted in both the pancreatic and the bile ducts (black arrow). Stenosis was also noted in the middle bile duct, suggesting bile duct cancer (white arrow). The yellow arrows were the stump of the bile duct.

**Figure 4:**
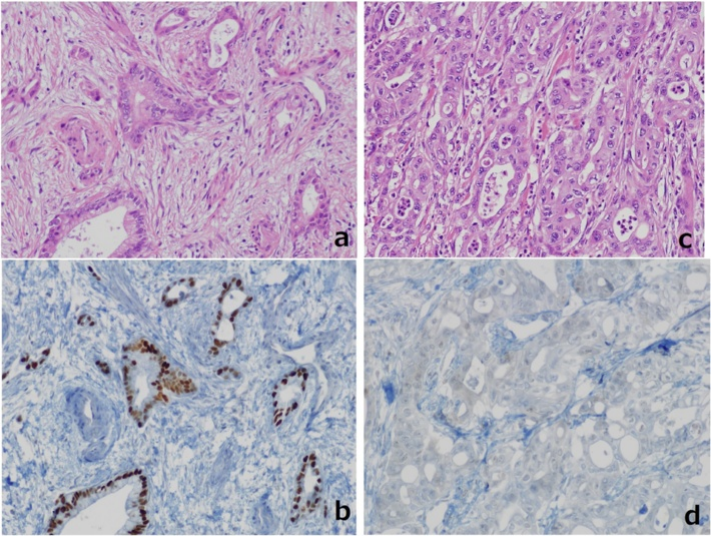
The pancreatic head tumor and the tumor at the confluence of the cystic duct. Pathologic examination revealed a well-differentiated tubular adenocarcinoma in the bile duct **(a)** (H.E., ×40), which was p53-positive **(b)**, and a moderately differentiated tubular adenocarcinoma in the pancreatic duct **(c)** (H.E., ×40), which was p53-negative **(d)**.

**Figure 5:**
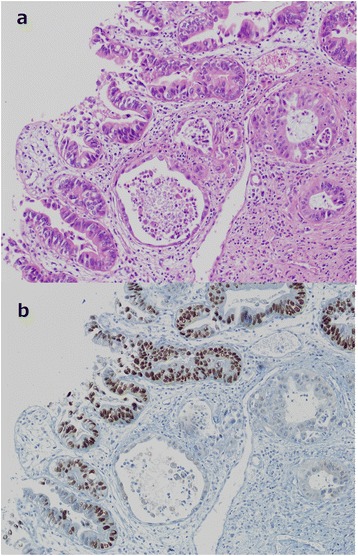
Two histologically different tumors observed in the head of the pancreas. Within the pancreatic parenchyma, there is a ductal structure with nuclear atypia **(a)** (H.E., ×40). Two types of carcinoma collide with each other in the pancreas **(b)** (immunostaining for p53).

**Figure 6:**
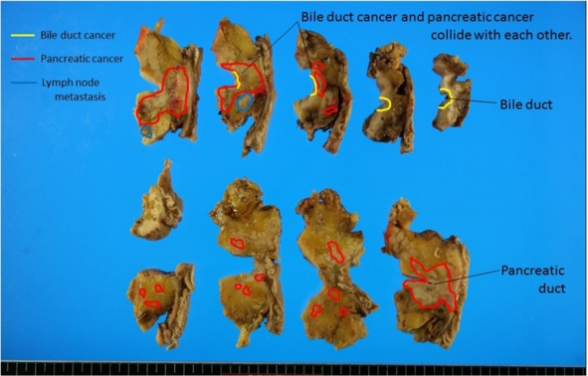
The collision between the pancreatic cancer and the bile duct cancer at the resection site. The pancreatic cancer and the bile duct cancer are colliding within the pancreatic head.

**Figure 7:**
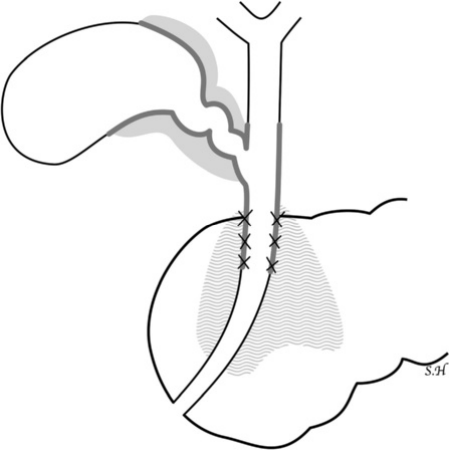
Pancreatic cancer and bile duct cancer colliding with the pancreatic head. The cholangiocarcinoma and the pancreatic head cancer collided at the X mark.

The resected specimen demonstrated tumors in both the confluence of the cystic duct and the pancreatic head (Figure 3). The tumor in the head of the pancreas was a moderately differentiated tubular adenocarcinoma (T3, N1, M0, stage II B) (UICC). The tumor at the confluence of the cystic duct was a well-differentiated tubular adenocarcinoma (T2, N0, M0, stage II). Immunostaining was conducted for the tumor markers mucin-1 (MUC1), mucin-2 (MUC2), mucin-5AC (MUC5AC), mucin-6 (MUC6), cytokeratin-7 (CK7), cytokeratin-19 (CK19), cytokeratin-20 (CK20), and tumor protein p53 (p53). For both tumors, MUC1, MUC5AC, CK7, and CK19 were positive, while MUC2 and CK20 were negative. However, the pancreatic head tumor was negative for p53 and MUC6, while these were both positive in the tumor at the confluence of the cystic duct (Figure 4). Because two histologically different tumors were observed in the head of the pancreas, these were diagnosed as collision cancer in which bile duct and pancreatic cancers collided (Figure 5). The mapping of the resected specimen showed pancreatic cancer and bile duct cancer colliding with the pancreatic head (Figures 6 and 7).

### Postoperative course

Gemcitabine chemotherapy was administered as adjuvant chemotherapy following standard treatment guidelines for pancreatic cancer. However, the patient died of multiple lung metastases 4 years and 1 month after surgery.

### Discussion

According to the criteria presented by Warren and Gates [2], synchronous malignant neoplasms are defined as follows: (1) each of the tumors must present a definite picture of malignancy; (2) each must be distinct; and (3) the possibility that one is a metastatic lesion caused by the other must be ruled out. Also, Moertel et al. [3] arbitrarily classified tumors diagnosed within a 6-month period as ‘simultaneous lesions’ and those diagnosed at intervals of longer than 6 months as ‘interval lesions.’

The number of reports of dual cancers is increasing every year due to the marked progress of diagnostic imaging technology in recent years, increasing frequency of medical checkups, and the aging of the population [4-6]. The reported rate of pancreatic cancer associated with other organ malignancies is 5.6% to 16.6% [7,8]. The ranking of cancers presenting concomitantly with pancreatic cancer, from the highest to the lowest incidence, is as follows: gastric cancer, thyroid cancer, lung cancer, and breast cancer.

Collision cancers refer to tumors located in the same organ or anatomical site. According to the World Health Organization (WHO) histological classification, collision tumors comprise at least two different malignant components, with no mixed or transitional area in between. Among intrapancreatic collision tumors, the incidence of collision between an intraductal papillary mucinous neoplasm and cancers of other types is high [9,10]. In the pancreatic head area, adenocarcinoma and other types of cancer have been reported; however, collision of two adenocarcinomas is extremely rare. To the best of our knowledge, this is the third reported case of a collision tumor between a bile duct adenocarcinoma and pancreatic adenocarcinoma.

The symptoms and imaging-based findings of pancreatic head cancer accompanied by bile duct infiltration are very similar to those of bile duct cancer accompanied by pancreatic infiltration, and it is difficult to distinguish between these two conditions before surgery in many cases. In our patient, a mass was palpated in the pancreatic head during surgery, and a hard, hypertrophic bile duct was also palpated at the confluence of the three ducts slightly distal to the pancreatic mass. In the resected specimen, a mass was also observed at the confluence of the three ducts slightly proximal to the mass in the pancreatic head, suggesting dual cancer. Obstructive jaundice associated with pancreatic head cancer is generally assumed to result from malignant invasion of the bile duct by the pancreatic cancer. However, in this patient, the stenosis of the ducts within the head of the pancreas and the hypertrophy of the bile duct at the confluence of the three ducts were slightly separated from each other. Thus, it is possible that this particular dual cancer might have been diagnosed earlier if endoscopic ultrasonography or intraductal ultrasonography had been performed.

The clinical behavior, associated prognosis, and malignant potential of collision tumors are generally related to the most aggressive of the colliding tumors [10]. Pancreatic carcinoma is associated with a very poor prognosis and a short survival period. Therefore, the prognosis of patients with dual cancers involving pancreatic cancer mainly depends on the prognosis associated with the pancreatic cancer [11]. On the other hand, Geng Ming Niu et al. [1] reported ten cases of collision cancer in which the colliding cancers were pancreatic and periampullary cancers, and the outcome was worse than that of patients with pancreatic cancer alone.

In reports of collision cancers in which periampullary carcinoids and adenocarcinomas collided, the difference in the origins of the tumors was observed on CEA staining [12,13]. Hirono et al. [9] also reported differences among the CK20, MUC2, and p53 expression levels on immunostaining of cancers presenting in the bile duct and the ampulla of Vater, showing that they were independent cancers colliding with each other rather than one type of cancer. In our patient, differences were noted in the expression levels of p53 and MUC6, showing that two histopathologically different cancers had collided with each other in the pancreatic parenchyma. No transition between the two cancers was noted, leading to a diagnosis of collision cancer composed of bile duct and pancreatic cancers.

## Conclusions

The pathological and immunohistochemical findings in this case support the assertion that two separate cancers, one originating from the bile duct and one from the head of the pancreas, collided with each other, and thus, this case can be defined as one of collision cancers. Our case report is the third report of a case of collision cancer between a bile duct cancer and a pancreatic cancer.

## Consent

Written informed consent was obtained from the patient for publication of this case report and any accompanying images. A copy of the written consent is available for review by the editor in chief of this journal.
